# Performance of Deep Learning Models in Forecasting Gait Trajectories of Children with Neurological Disorders

**DOI:** 10.3390/s22082969

**Published:** 2022-04-13

**Authors:** Rania Kolaghassi, Mohamad Kenan Al-Hares, Gianluca Marcelli, Konstantinos Sirlantzis

**Affiliations:** School of Engineering, University of Kent, Canterbury CT2 7NT, UK; m.k.al-hares@kent.ac.uk (M.K.A.-H.); g.marcelli@kent.ac.uk (G.M.); k.sirlantzis@kent.ac.uk (K.S.)

**Keywords:** deep learning, forecasting, prediction, gait, children, kinematics, motion capture, exoskeletons, artificial intelligence, actuated orthoses, pathological gait, lower limb robot

## Abstract

Forecasted gait trajectories of children could be used as feedforward input to control lower limb robotic devices, such as exoskeletons and actuated orthotic devices (e.g., Powered Ankle Foot Orthosis—PAFO). Several studies have forecasted healthy gait trajectories, but, to the best of our knowledge, none have forecasted gait trajectories of children with pathological gait yet. These exhibit higher inter- and intra-subject variability compared to typically developing gait of healthy subjects. Pathological trajectories represent the typical gait patterns that rehabilitative exoskeletons and actuated orthoses would target. In this study, we implemented two deep learning models, a Long-Term Short Memory (LSTM) and a Convolutional Neural Network (CNN), to forecast hip, knee, and ankle trajectories in terms of corresponding Euler angles in the pitch, roll, and yaw form for children with neurological disorders, up to 200 ms in the future. The deep learning models implemented in our study are trained on data (available online) from children with neurological disorders collected by Gillette Children’s Speciality Healthcare over the years 1994–2017. The children’s ages range from 4 to 19 years old and the majority of them had cerebral palsy (73%), while the rest were a combination of neurological, developmental, orthopaedic, and genetic disorders (27%). Data were recorded with a motion capture system (VICON) with a sampling frequency of 120 Hz while walking for 15 m. We investigated a total of 35 combinations of input and output time-frames, with window sizes for input vectors ranging from 50–1000 ms, and output vectors from 8.33–200 ms. Results show that LSTMs outperform CNNs, and the gap in performance becomes greater the larger the input and output window sizes are. The maximum difference between the Mean Absolute Errors (MAEs) of the CNN and LSTM networks was 0.91 degrees. Results also show that the input size has no significant influence on mean prediction errors when the output window is 50 ms or smaller. For output window sizes greater than 50 ms, the larger the input window, the lower the error. Overall, we obtained MAEs ranging from 0.095–2.531 degrees for the LSTM network, and from 0.129–2.840 degrees for the CNN. This study establishes the feasibility of forecasting pathological gait trajectories of children which could be integrated with exoskeleton control systems and experimentally explores the characteristics of such intelligent systems under varying input and output window time-frames.

## 1. Introduction

Assistive devices such as wheelchairs and exoskeletons are designed to facilitate or enhance movement [1]. For people with impaired motor or nervous systems which affect their movement, these devices increase mobility and independence and allow for better interaction with the environment [1].

Exoskeletons are external load-bearing robotic suits made of sensors, actuators, and controllers to increase the strength and endurance of the user [2,3]. In the 1960s, after earlier conceptualisations, the first exoskeleton ’Hardiman’ was built by General Electric [2]. Since then, exoskeletons have witnessed continuous advancements in their structure and control [3].

Exoskeletons can be divided into two broad categories: passive and active. Passive exoskeletons are purely mechanical devices that provide torque to joints when contractible materials such as springs and dampeners expand, releasing the stored energy [4]. On the other hand, active exoskeletons contain actuators that provide torque to joints using an external power supply [4].

There are exoskeletons designed for the upper limbs, lower limbs, or the full body [5]. Lower limb exoskeletons are often used for three primary applications [6]. Firstly, they are used for the rehabilitation of the gait of patients with mobility disorders, the benefits of which extend to the therapists, relieving them from the strain of fully handling the patients themselves [6]; examples include Robotic Orthosis Lokomat by Hocoma [7], Ekso GT exoskeleton by Ekso Bionics [8], and ALEX by the University of Delware [9]. Secondly, exoskeletons can be used by patients with more severe mobility disorders that are partially or completely paralysed, outside rehabilitation centres [6]; they can assist with their daily locomotion, allowing them to stand/walk for longer periods. This has reportedly led to desirable physiological and psycho-somatic outcomes, including but not limited to improved spasticity, quality of life, bowel function, etc. [10]; the Vanderbilt exoskeleton is one example [11]. Thirdly, exoskeletons are used to augment the abilities of healthy users, allowing heavy loads to be carried with more ease or a movement to be performed with lower strain [6]; examples include Berkeley Lower Extremity Exoskeleton (BLEEX) [12] and Hybrid Assistive Limb (HAL) exoskeleton [13].

Powered exoskeletons need a control strategy to guide the exoskeleton’s interaction with the user. The control strategy coordinates the exoskeleton’s movement with the user’s body when full support is provided, while it synchronises the exoskeleton’s movement with the user’s body when partial support is provided [14]. The control strategy often consists of a three-level hierarchy: high, mid, and low levels [14]. The high level of control is responsible for intention detection, predicting what state the user would like to be in and the overall behaviour of the exoskeleton [14,15,16]. This includes estimating desired torque from EMG signals [17], classifying locomotion modes (e.g., standing up, sitting down, walking up/down a staircase, etc.) [18] and their transitions, as well as environment classification (predicting the user’s interaction with the surrounding environment [19]). The mid-level control is responsible for selecting one of the continuous states of the exoskeleton and switching between them [14,15]. Baud et al. [14], in their review on control strategies for lower limb exoskeletons, divide this level into two sub-levels: detection/synchronisation and action. Detection/synchronisation involves identifying the state of the user, such as the phase of gait, while action is responsible for computing the output of the identified state. Lastly, the low-level control directly controls the actuators and is responsible for implementing the control strategy to supply torque to the joints. It tracks the reference input and ensures stability [15,20,21].

The timing and magnitude of the supportive torque provided depend on the control strategy implemented, which is highly influenced by the exoskeleton’s application. In rehabilitation applications, an exoskeleton may follow a predefined trajectory. This is referred to as *trajectory tracking* and the trajectory tracked can be the trajectory of a healthy user [6]. Another control strategy used in exoskeletons for rehabilitation is *assist as needed*, where the amount of support provided by the exoskeletons is variable and may change through the course of rehabilitation. Impedance control is an example of an *assist as needed* strategy, where the assistance provided depends on the effort of the patient [6]. In locomotion assistance applications, trajectory tracking is also most commonly used. The trajectory tracked could be a predefined trajectory for a healthy user, or in the case of hemiparetic patients, the trajectory used for the pathological limb can be the trajectory of the healthy limb (also known as complementary limb motion estimation [22]). As for strength augmentation applications, hybrid and force control are commonly used [6].

Gait trajectory prediction can be integrated into the control strategy of exoskeletons [23]. Several researchers investigated the use of deep learning for gait trajectory prediction. Liu et al. [24] developed a deep spatio-temporal model that consists of Long Short-Term Memory (LSTM) units to predict two time-steps in the future, with predictions averaged to smooth fluctuations. Zaroug et al. [25] implemented an auto-encoder LSTM for predicting linear acceleration and angular velocity trajectories. They experimented with varying lengths of input time-steps, between five and 40 steps, to predict five or 10 steps in the future (equivalent to 30 ms or 60 ms). An LSTM with a weighted discount loss function was proposed by Su et al. [26] for the prediction of the angular velocities of thigh, shank, and foot segments. They used 10 or 30 time-steps as input to predict five or 10 steps in the future, corresponding to 100 ms and 200 ms, respectively. Hernandez et al. [27] used a hybrid Convolutional Neural Network (CNN) and LSTM neural network, DeepConvLSTM, to forecast kinematic trajectories with an average MAE of 3.6${}^{\circ }$, while Jia et al. [28] implemented a deep neural network with LSTM units and a feature fusion layer that combines kinematic (i.e., joint angles) and physiological (i.e., EMG) data for trajectory prediction. Zarough et al. [29] also compared between vanilla, stacked, bidirectional, and autoencoder LSTMs while Zhu et al. [30] used attention-based CNN-LSTM, predicting trajectories 60 ms in the future.

Values of kinematic parameters within a gait cycle vary more significantly between different individuals (inter-subject) compared to the values of kinematic parameters of the same individual (intra-subject) [31]. Intra-subject trajectory prediction models (models tested on data from the same individuals used for training the models) have been shown to be more accurate in their predictions than inter-subject models (models tested on data from individuals who were not used for training the models) [26]. However, the variability of pathological gait compared to healthy gait is even greater. For example, children with spastic cerebral palsy were found to have higher within-day and between-day variability compared to healthy children, possibly attributed to the spasticity that limits the range of motion of their joints [32,33].

Given that existing studies have used models trained on healthy gait trajectories only, the ability of deep learning models to forecast pathological trajectories with greater heterogeneity and variability is yet to be evaluated. The main contribution of this study is to investigate, for the first time, the performance of deep learning networks, specifically the Long Short-Term Memory (LSTM) neural network and Convolutional Neural Network (CNN), in forecasting pathological gait trajectories of children with neurological disorders. We conduct a comparison between the two networks. Furthermore, we investigate the influence of the length of the input and output windows on prediction accuracy and provide technical recommendations.

## 2. Materials and Methods

### 2.1. Data

The deep learning models implemented in our study are trained on data from children with neurological disorders. The data used are available online and were collected by Gillette Children’s Speciality Healthcare over the years 1994–2017 [34]. They have been previously used for the development of a deep learning model to automatically detect gait events, specifically foot contact and foot off events [35]. The children of the dataset ranged from 4 to 19 years of age. The majority of them had cerebral palsy (73%), while the rest had a combination of neurological, developmental, orthopaedic, and genetic disorders (27%). Children were recorded with a motion capture system (VICON) with a sampling frequency of 120 Hz while walking for 15 m. The data consist of a 99-dimensional vector with kinematics and marker positions. The data were divided into a training and testing set. The statistical distribution of the features of the children in the training set is as follows: age (11.4 ± 6.2 years), weight (35.7 ± 17.7 kg), height (135.7 ± 21.6 cm), leg length (70.3 ± 14.0 cm), and walking speed (0.84 ± 0.28 m/s). The statistical distribution of the children in the testing set is as follows: age (11.0 ± 4.5 years), weight (35.9 ± 16.7 kg), height (135.6 ± 21.4 cm), leg length (70.6 ± 12.8 cm), and walking speed (0.85 ± 0.29 m/s) [35]. In our study, only kinematics were used for trajectory forecasting; these include angles of the hip, knee, and ankle in the yaw, pitch, and roll dimensions. The kinematics represent Euler angles, calculated using the plug-in-gait mechanical model [35].

### 2.2. Data Processing

Euler angles of the hip, knee, and ankle in yaw, pitch, and roll dimensions were available for both legs, however, only one leg was used for training the models. The pre-processing of data involved trimming the leading and the trailing zeros, and the removal of trails with spurious data (which were assumed to be Euler angles greater or less than a 90${}^{\circ }$ cut-off).

**Figure 1:**
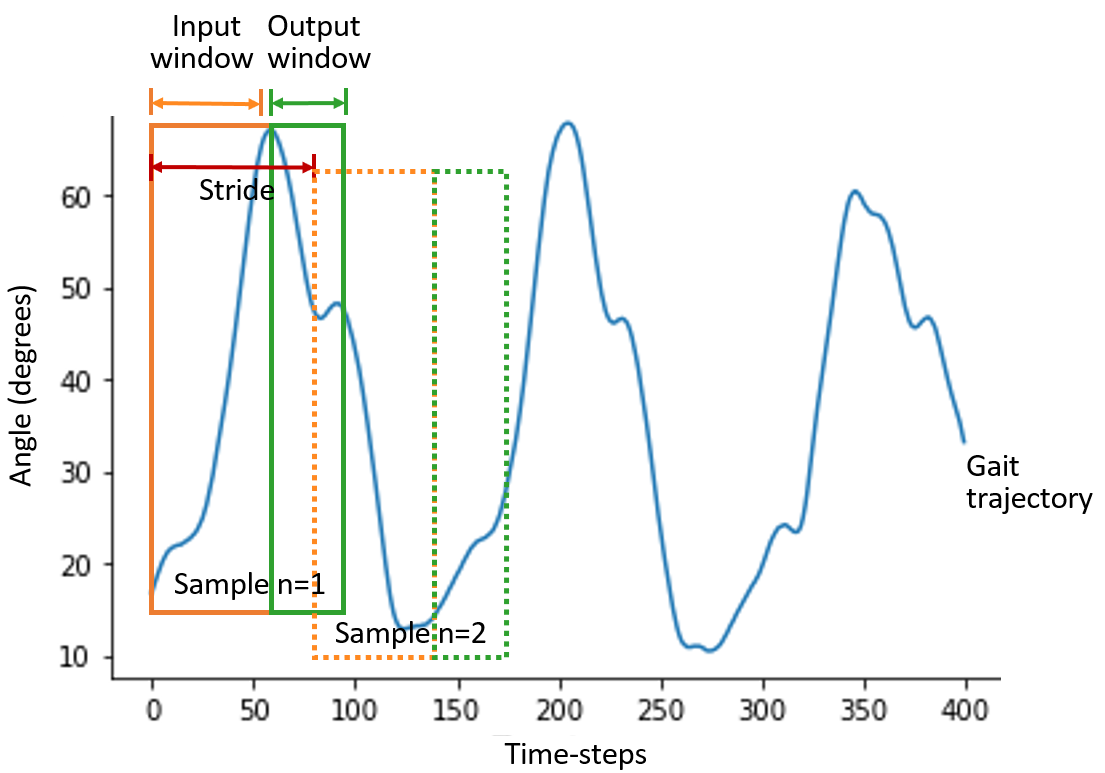
Illustration of the sliding window method. Continuous gait trajectories are used to generate input and output windows for training the model using the sliding window method. Each pair of input and output windows forms one sample. Several samples can be generated from one continuous gait trajectory by sliding the window for a specified distance, also known as stride length.

Since deep learning models are required to be trained with fixed-length sequences, fixed-length inputs and their corresponding target trajectories were generated using the sliding window method, illustrated in Figure 1. The sliding window method involves generating a shorter sequence $x_{in}$ with *k* time-steps, where *k* is the size of the input, i.e., the number of time-steps utilised by the model to make predictions, $x_{in}=\left\{x_{1},x_{2},\cdots ,x_{k}\right\}$. Another shorter sequence $y_{out}$ with *z* time-steps is created, were *z* is the size of the output, i.e., the number of time-steps that will be predicted by the model, $y_{out}=\left\{x_{k+1},x_{k+2},\cdots ,x_{k+z}\right\}$. Each input window has a corresponding output window (target label to train the models), and the two windows represent one training sample. The stride, which is the distance between the beginning of one sample and the beginning of the next sample, is set to 5 time-steps. Ten samples are generated from each trial.

The models were trained using several sizes of input and output windows. The input window sizes for the LSTM were 50, 100, 200, 400, 600, 800, and 1000 ms. Given that the data were captured at a sampling frequency of 120Hz, those durations correspond to 6, 12, 24, 48, 72, 96, and 120 time-steps. Output window sizes for the LSTM were 8.33, 25, 50, 100, and 200 ms which correspond to 1, 3, 6, 12, and 24 time-steps. Every combination of input and output window sizes was used, yielding a total of 35 combinations. As for the CNN, we only focused on using 6 and 120 input time-steps (the smallest and largest input sizes we used with the LSTM) to predict 1, 3, 6, 12, and 24 output time-steps. We used these time ranges following values proposed in the literature by researchers that forecasted healthy gait. Zaroug et al. experimented with a wide range of input windows from 5–40 steps to forecast 5 or 10 steps in the future, which correspond to 30 and 60 ms respectively [25]. Output windows ranging from 50–60 ms were common too [25,28,30]. A few researchers have also predicted output windows of 100 ms or larger [26,29].

For *n* training samples, *f* features (hip, knee, and ankle angles in the yaw, pitch, and roll directions), $l_{in}$ input window size and $l_{out}$ output window size, an input matrix *X* and target output matrix *Y* are created, where $X\in R^{n\times l_{in}\times f}$ and $Y\in R^{n\times l_{out}\times f}$. The matrices are then normalised such that X∈[0,1] and Y∈[0,1]. The aim is to build a model g() that maps *X* to $\hat{Y}$, i.e., $\hat{Y}=g\left(X\right)$, where $\hat{Y}$ is a close approximation to true value *Y*.

### 2.3. Long Short-Term Memory (LSTM) Architecture

The Long Short-Term Memory (LSTM) neural network is commonly used for time-series applications including forecasting future trajectories. It is a type of gated Recurrent Neural Network (RNN), which solved the issue of vanishing and exploding gradients during training and is capable of learning long-term dependencies [36]. Each LSTM cell contains three gates: input, output, and forget gate. The equations of these gates, as explained in Goodfellow et al. [36], are reported below.

The forget gate unit $f_{i}^{\left(t\right)}$ equation for time-step *t*, cell *i*, input vector $x^{\left(t\right)}$, hidden layer vector $h^{\left(t\right)}$, biases $b^{f}$, recurrent weights $W^{f}$, and input weights $U^{f}$ is:

(1) $$f_{i}^{\left(t\right)}=\sigma \left(b_{i}^{f}+\sum_{j}U_{i,j}^{f}x_{j}^{\left(t\right)}+\sum_{j}W_{i,j}^{f}h_{j}^{\left(t-1\right)}\right).$$

The internal state of an LSTM cell, $s_{i}^{\left(t\right)}$, is updated depending on the value of the forget gate unit $f_{i}^{\left(t\right)}$, and its equation for biases *b*, input weights *U*, and recurrent weights *W* is:

(2) $$s_{i}^{\left(t\right)}=f_{i}^{\left(t\right)}s_{i}^{\left(t-1\right)}+g_{i}^{\left(t\right)}\sigma \left(b_{i}+\sum_{j}U_{i,j}x_{j}^{\left(t\right)}+\sum_{j}W_{i,j}h_{j}^{\left(t-1\right)}\right).$$

The external input gate unit, $g_{i}^{\left(t\right)}$, is calculated as:

(3) $$g_{i}^{\left(t\right)}=\sigma \left(b_{i}^{g}+\sum_{j}U_{i,j}^{g}x_{j}^{\left(t\right)}+\sum_{j}W_{i,j}^{g}h_{j}^{\left(t-1\right)}\right).$$

The output of the LSTM cell $h_{i}^{\left(t\right)}$ is calculated as:

(4) $$h_{i}^{\left(t\right)}=tanh\left(s_{i}^{\left(t\right)}\right)q_{i}^{\left(t\right)}.$$

For biases $b^{o}$, input weights $U^{o}$, and recurrent weights $W^{o}$, the value of the output gate unit is:

(5) $$q_{i}^{\left(t\right)}=\sigma \left(b_{i}^{o},+\sum_{j}U_{i,j}^{o}x_{j}^{\left(t\right)}+\sum_{j}W_{i,j}^{o}h_{j}^{\left(t-1\right)}\right).$$

**Figure 2:**
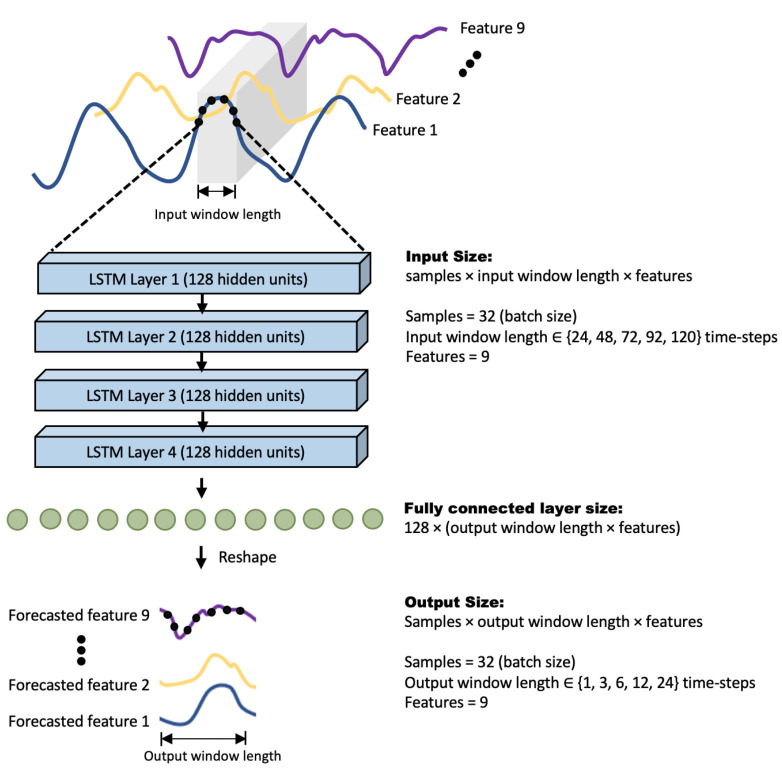
Architecture of the LSTM network used in this study.

The LSTM network implemented in this study contained 4 layers of LSTM units, with 128 units per layer. The hidden state of the final layer is used as input to a fully connected layer which is then reshaped to obtain the desired output. The overall architecture is illustrated in Figure 2.

### 2.4. Convolutional Neural Network (CNN) Architecture

In this study, a Convolutional Neural Network (CNN) is used to map input trajectories *X* to forecasted predictions $\hat{Y}$. The convolution operation is formed between a sequence and a kernel, whose weights are tuned during the learning process. Equation (6) is used to calculate the output of the convolution operation *S*, adapted from the format included in Goodfellow et al. [36] to work with a 1D time-series *I* and kernel *K*.

(6) $$S\left(i\right)=\left(I*K\right)\left(i\right)=\sum_{m}I\left(m\right)K\left(i-m\right).$$

**Figure 3:**
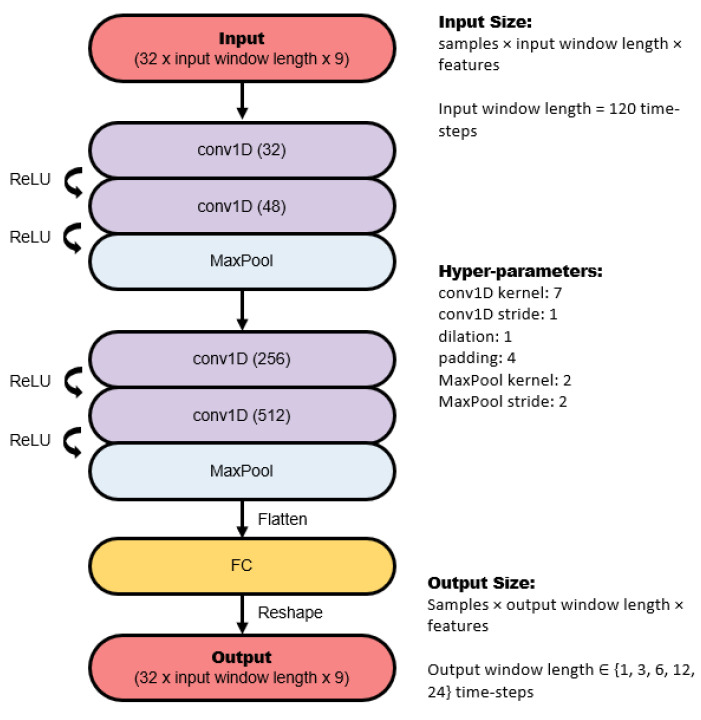
Architecture of the convolutional neural network used in this study.

The CNN architecture consists of several 1D convolutions and pooling layers, followed by a dense fully connected layer. The number of kernels in each convolution layer, as well as the size of the pooling layers, is illustrated in Figure 3. Dilated convolutions were tried, but they did not improve performance, therefore they were not included.

### 2.5. Baseline Methods

The performance of the deep learning models was benchmarked against a simpler machine learning model, a Fully Connected Network (FCN), and two non-intelligent models, which we refer to as Naïve Method 1 and Naïve Method 2. The FCN contains three hidden layers and 200 nodes per layer. As for the non-intelligent models, the first naïve method uses the final time-step in the input window as the predicted value for all output time-steps. The second naïve method uses the mean value of the input as the predicted value for all output time-steps.

### 2.6. Details of Network Implementation

The objective of the deep learning models is to find a mapping between the input trajectories *X* and forecasted trajectories $\hat{Y}$, such that the error between predicted trajectories $\hat{Y}$ and true trajectories *Y* is minimised. The loss function that has been used to optimise the deep learning models is the Mean Squared Error (MSE).

The number of trials used from the dataset was 16,782. Each trial was used to extract 10 samples using the sliding window method, described in Section 2.2, resulting in a total of 167,820 samples. The samples were split into training (70%), validation (20%), and testing (10%) sets. Both the CNN and LSTM are trained in mini-batches, where the size of each batch is 32 samples, with the Adam optimiser used for learning.

To select the hyper-parameters and architecture for the CNN, LSTM, and FCN models (described in Section 2.3, Section 2.4 and Section 2.5), a hyper-parameter search was performed. Hyper-parameter optimisation involved defining a search space, where windows of values of some parameters of the model were chosen (e.g., learning rate, number of layers of LSTM, number of hidden units, and size of kernels). The objective is to choose the value of parameters that optimises the performance of the networks. The tree-structured Parzen estimator algorithm, which is a type of Bayesian hyper-parameter sampler [37], was used to select the optimal parameters (from a defined search space) that minimise the validation loss. The search space for the hyper-parameters and the corresponding selected values are included in Table 1. The parameters were optimised for predictions with an input window size of 72 time-steps, and an output window size of 12 time-steps. The number of epochs was also a parameter that was included in the hyper-parameter optimisation, but the values were fine-tuned manually afterwards. The numbers of epochs for training the LSTM, CNN, and FCN were 60, 150, and 180, respectively. Once the optimal architecture and parameters of the networks were selected, the training and validation sets were combined to train these networks. The total numbers of trainable parameters of the optimised LSTM, CNN, and FCN are 495,320, 4,666,888, and 380,216, respectively. The final performance of the networks was evaluated on the testing set.

The framework described has been implemented using Python, with the following libraries: Pytorch, Numpy, Matplotlib, SciPy, and Scikit-learn. Optuna was used for the hyper-parameter search [37]. An Nvidia Geforce RTX 2070 GPU was also used for computation.

### 2.7. Evaluation Metrics

Several metrics have been used to evaluate the performance of the models. To measure how close the predicted trajectories $\hat{Y}$ are to the observed trajectories *Y* we have calculated the mean square error MSE, mean absolute error MAE, and Pearson correlation coefficient *P*. These were calculated after the de-normalisation (i.e., re-scaling to the original ranges) of the predicted trajectories. Given that *n* is the number of testing samples, *f* is the number of features, and $l_{out}$ is the output window size, the equations are as follows:

Mean squared error:

(7) $$MSE=\frac{1}{n\times f\times l_{out}}\sum_{i=1}^{n}\sum_{j=1}^{f}\sum_{k=1}^{l_{out}}{\left(y_{i,j,k}-{\hat{y}}_{i,j,k}\right)}^{2}.$$

Mean squared error standard deviation:

(8) $$\sigma_{MSE}=\sqrt{\frac{1}{n\times f\times l_{out}}\sum_{i=1}^{n}\sum_{j=1}^{f}\sum_{k=1}^{l_{out}}\left({\left(y_{i,j,k}-{\hat{y}}_{i,j,k}\right)}^{2}-MSE\right)}.$$

Mean absolute error:

(9) $$MAE=\frac{1}{n\times f\times l_{out}}\sum_{i=1}^{n}\sum_{j=1}^{f}\sum_{k=1}^{l_{out}}|y_{i,j,k}-{\hat{y}}_{i,j,k}|.$$

Mean absolute error standard deviation:

(10) $$\sigma_{MAE}=\sqrt{\frac{1}{n\times f\times l_{out}}\sum_{i=1}^{n}\sum_{j=1}^{f}\sum_{k=1}^{l_{out}}\left(|y_{i,j,k}-{\hat{y}}_{i,j,k}|-MAE\right)}.$$

Pearson correlation coefficient:

(11) $$P=\frac{1}{f}\sum_{j=1}^{f}\frac{cov(y_{j},{\hat{y}}_{j})}{std\left(y_{j}\right)\times std\left({\hat{y}}_{j}\right)}.$$

These metrics are used to evaluate and compare the performance of the networks we implemented, with results presented in Section 3.

## 3. Results

### 3.1. LSTM Network Performance for Varying Input and Output Window Sizes

The LSTM model has been trained using 35 combinations of input and output window sizes. The input window sizes were 6, 12, 24, 48, 72, 96, and 120 time-steps, which correspond to 50, 100, 200, 400, 600, 800, and 1000 ms, respectively, while the output window sizes were 1, 3, 6, 12, and 24 time-steps, and correspond to 8.33, 25, 50, 100, and 200 ms. The performance of the models (MAE, MSE, and Pearson correlation coefficient) are reported in Table 2.

**Figure 4:**
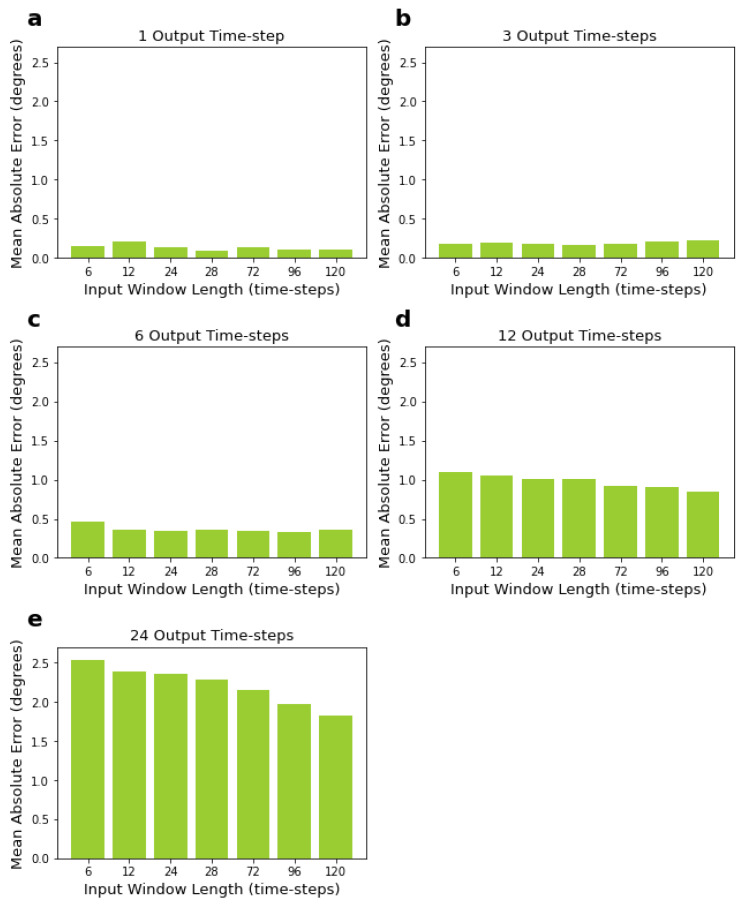
Performance of LSTM, measured by MAE, for varying input and output window sizes. Input sizes range from 6 to 120 time-steps, corresponding to 200 to 1000 ms. Output sizes range from 1 to 24 time-steps, corresponding to 8.33 to 200 ms. Sub-figures (**a**–**e**) correspond to networks with 1, 3, 6, 12, and 24 output time-steps, respectively.

Results in Table 2 show that the smaller the size of the output window, the lower the error of predictions. LSTMs predicting one output time-step had the lowest mean errors, while LSTMs predicting 24 time-steps had the highest mean errors, as expected. On the other hand, the size of the input window did not significantly influence the mean losses when predicting short output windows, specifically six output time-steps or below (see Figure 4a–c). The size of the input window had an influence when predicting larger output windows, i.e., 12 and 24 time-steps, with larger input sizes resulting in lower mean errors (see Figure 4d,e). For 12 and 24 time-step output windows, using 120 time-steps as input window size, which is the largest input size we tried, led to the lowest mean absolute errors (see Figure 4d,e).

### 3.2. Performance of the CNN and Comparisons with LSTM Network

We trained the CNN and compared its performance with the LSTM network. We only focused on using six and 120 input time-steps (the smallest and largest input sizes we used with the LSTM) to predict 1, 3, 6, 12, and 24 output time-steps. The performances of both models (MAE, MSE, and Pearson correlation coefficient) are reported in Table 3.

**Figure 5:**
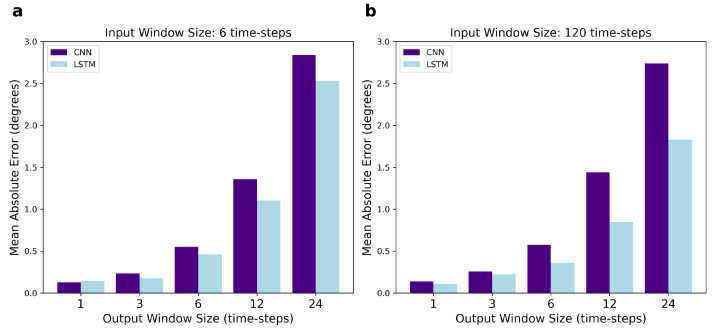
Comparison of the performance of the CNN and LSTM network, measured by MAE, for varying output window sizes. In (**a**), the input size is fixed at 6 time-steps, while in (**b**) the input is fixed at 120 time-steps.

As shown in Table 3, the CNN errors increase when increasing the number of predicted time-steps, as observed with LSTM. In Figure 5, we compare the performance of the CNN and LSTM and it is noticeable that their mean errors are very similar when predicting small output windows, such as one and three time-steps. However, the difference in the MAE for CNN and LSTM widens for larger output windows including 6, 12, and 24 future time-steps, with LSTM outperforming CNN. Interestingly, the size of this difference depends on the input window size: when six time-steps are used as input, the CNN MAE is larger than the LSTM one; when 120 time-steps are used as input, the difference in their MAEs is even larger.

### 3.3. Benchmarking Performance of Deep Learning Models

We benchmark the performance of our two deep learning models to a simpler machine learning architecture, the Fully Connected Network (FCN), and two naïve/non-intelligent methods. The first naïve method uses the final time-step in the input window as the predicted value for all output time-steps. The second naïve method uses the mean value of the input as the predicted value for all output time-steps. Table 4 shows the MAE for the deep learning and benchmark models. Our deep learning models outperformed all naïve methods and the majority of the FCN predictions. In two cases, the FCN obtained better results: the first was when the input and output window sizes were six and 24 time-steps, respectively, where the FCN had lower MAE compared to the LSTM and CNN; the other case was when the input and output sizes were 120 and 24 time-steps, respectively, with FCN performing better than CNN, but not better than LSTM.

Naïve Method 1 resulted in lower MAEs compared to Naïve Method 2, therefore, pairwise *t*-tests were conducted between the Naïve Method 1 and all other intelligent methods (LSTM, CNN, and FCN). This was done to determine whether the mean absolute errors were significantly different (with *p* < 0.05); the results in Table 4 confirm that intelligent models perform better than non-intelligent models. Furthermore, pairwise *t*-tests were conducted between the LSTM and all the other models; the differences in the MAEs were found to be statistically significant, as shown in Table 4.

### 3.4. Accuracy of the Models across the Different Time-Steps

**Figure 6:**
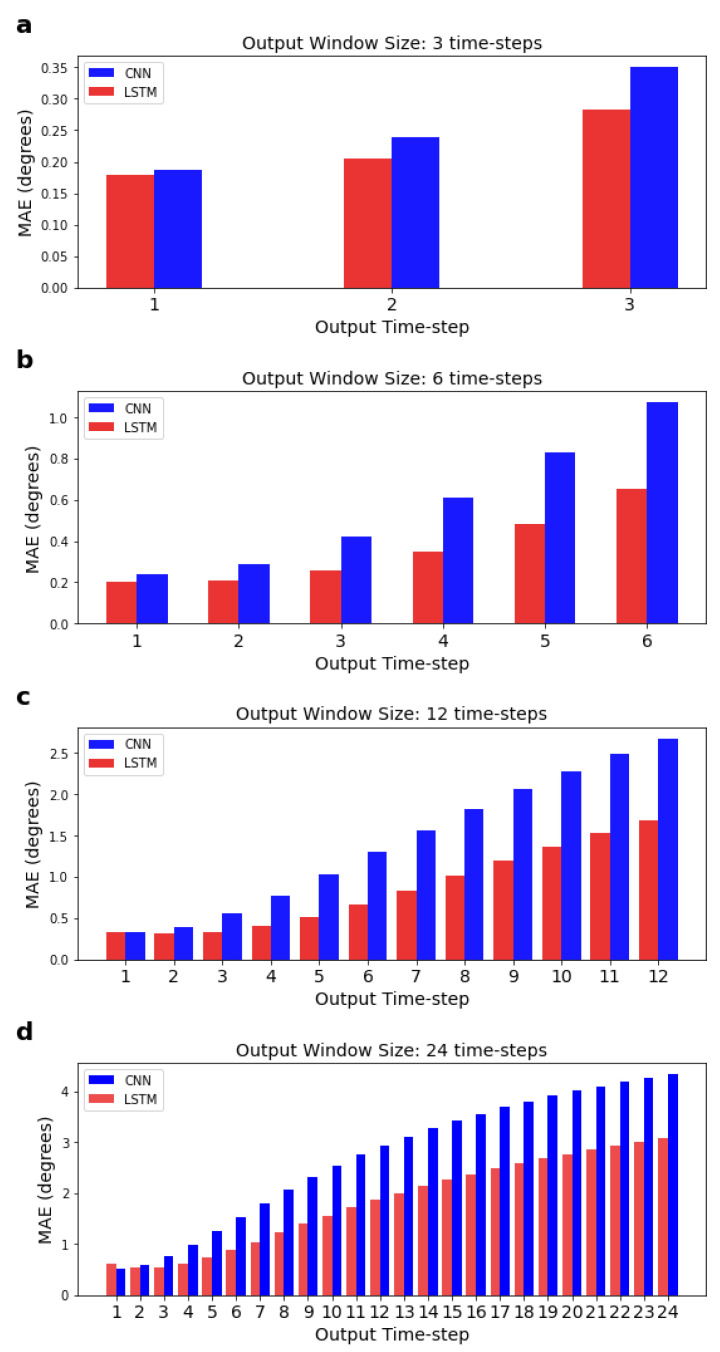
Mean absolute errors for each individual time-step predicted by the LSTM and CNN for a given output window. Input window size is fixed at 120 time-steps. Sub-figures (**a**–**d**) correspond to networks with 3, 6, 12, and 24 output time-steps, respectively.

**Figure 7:**
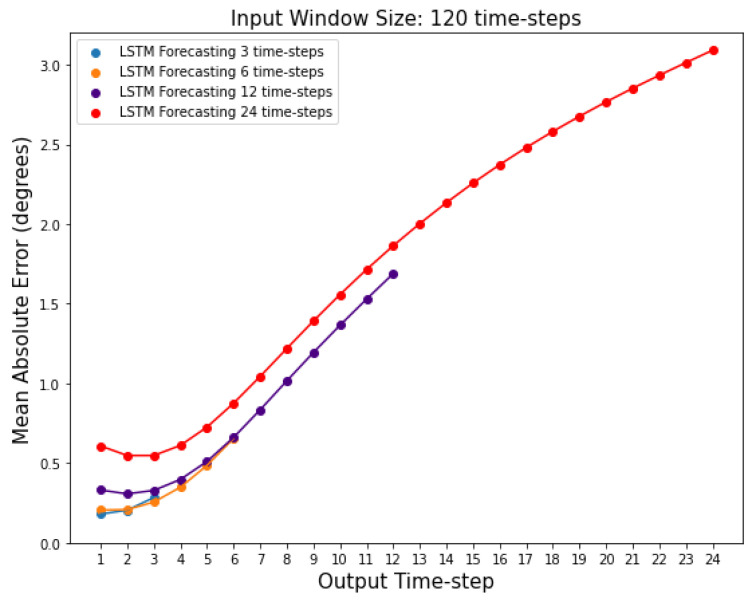
Mean absolute errors for each individual time-step predicted by the LSTM networks with 3, 6, 12, and 24 output window sizes. Input window size is fixed at 120 time-steps.

In the previous sections, we were reporting the mean error across all features and time-steps. Here, we calculate the MAE, for each time-step for a given output window, separately (see Figure 6); the MAE is calculated using an adapted form of Equation (9) (see Section 2.7), where the summation over *k* = 1:$l_{out}$ is not performed. As expected, results show that predictions further in the future deviate more from the actual values, and this deviation is more pronounced after around the 3rd time-step, as shown in Figure 7. Figure 7 also shows that the LSTM MAE increases with the increase in the size of the output window.

### 3.5. Performance of the Models for Each Joint

**Figure 8:**
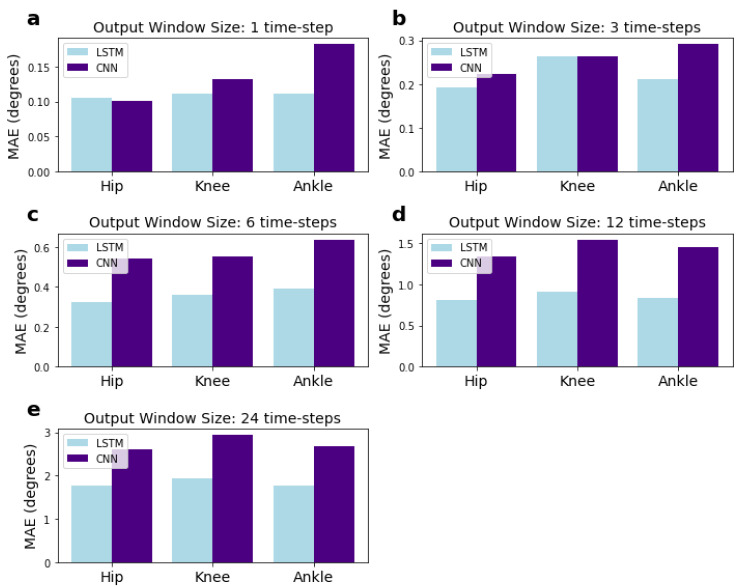
MAEs for each of the hip, knee, and ankle joints for the CNN and LSTM network with varying output sizes. Input window size is fixed at 120 time-steps and the MAE for each joint represents the combined MAE for the yaw, pitch, and roll dimensions. Sub-figures (**a**–**e**) correspond to networks with 1, 3, 6, 12, and 24 output time-steps, respectively.

We investigated whether errors for a particular joint were higher than others. The MAE results for each of the hip, knee, and ankle joints are presented in Figure 8. The MAE for each joint represents the combined errors for the angles predicted in the pitch, roll, and yaw dimensions. They were calculated using an adapted form of Equation (9) (see Section 2.7), where the numbers of features, *f*, in the summation over *j* = 1:*f* are reduced to the pitch, yaw, and roll angles for a single joint, rather than for all joints. The results do not show any particular trend.

## 4. Discussion

In this paper, we implement deep learning models, specifically LSTM and CNN, to forecast trajectories of children with pathological gait. To the best of our knowledge, this is the first time trajectories of pathological gait of children, which exhibit larger inter- and intra-subject variability compared to the trajectories of typically developing children, are predicted using deep learning models.

The advantage of deep learning models is that they make predictions based on current input data, but also utilise knowledge of learned representations of gait trajectories acquired during a prior learning stage from numerous gait sequences. We used LSTM and CNN to forecast hip, knee, and ankle trajectories based on varying input and output window sizes. Input window sizes for the LSTM were 50, 100, 200, 400, 600, 800, and 1000 ms (for data captured at a sampling frequency of 120Hz, these durations correspond to 6, 12, 24, 48, 72, 96, and 120 time-steps). Input window sizes for the CNN were 50 and 1000 ms (corresponding to six and 120 time-steps). The reason for using input window sizes up to 1000 ms is that the average length of a gait cycle for a typically developing school-aged child is 980–990 ms [38]. This means we trained deep learning models to make predictions based on data from approximately one full gait cycle, or lower. Output window sizes for the LSTM and CNN were 8.33, 25, 50, 100, and 200 ms (corresponding to 1, 3, 6, 12, and 24 time-steps); this means that we have tried to forecast up to 20% of the cycle. We used these time ranges following values proposed in the literature by researchers that forecasted healthy gait (refer to Section 2.2 for details).

The LSTM (a type of gated recurrent network) was selected for forecasting gait trajectories as it has been commonly and successfully used with sequential data [36]. The LSTM has the advantage of taking into account the order of values in an input sequence. It has the ability to learn long-term dependencies [36]. The LSTM network was compared to a CNN, which is mostly used for computer vision problems with 2D grid-like topology inputs using 2D convolutions, but it is increasingly used with time-series sequences using 1D convolutions [39]. Therefore, we implemented a CNN to evaluate if it shows promising performance in the task of forecasting trajectories for children with neurological disorders.

Our results show that the LSTM’s performance is better than the CNN. However, the difference between the MAE of the two networks was larger with larger input and output window sizes. The performance gap, measured in MAE, was highest with 120 time-steps as input, and 24 time-steps as output, and was 0.91 degrees. There was one case where the MAE for the CNN was higher than the LSTM, which used the smallest combination of input and output windows (six and one time-steps, respectively). For this case, the difference in MAE between the CNN and LSTM was small, 0.014 degrees, and the CNN had a higher standard deviation. Our results are different from those of Moreira et al. [40], who found that the CNN was more robust for ankle joint torque estimation based on kinematics, speed, and anthropometry. Our results are also different from those of Molinaro et al. [41], in which a temporal convolution network (with dilated convolution layers) outperformed an LSTM implementation. It must be stressed that Molinaro et al. considered joint moments rather than joint angles.

We also compared the influence of the size of the input and output windows on predictions. The size of the input window did not have a significant influence on the accuracy of the LSTM network when predicting small output window sizes (including one, three, and six time-steps). However, for predicting longer output window sizes (including 12 and 24 time-steps), larger input windows resulted in lower errors. For both 12 and 24 time-steps, the lowest error was achieved using 120 input time-steps. This is different from what was reported by [25], who found that after increasing input size beyond 30 time-steps, the mean errors for predicting five time-steps in the future increased (10 time-steps correspond to 60 ms in that paper).

There were cases in our results in which the difference between the actual and predicted trajectories was large compared to the mean absolute error. We could not tell whether this was due to the model’s lack of generalisability for certain types of pathological gait patterns, or due to an underlying issue with the data for those samples, such as sensor or marker errors. This is because the subjects were anonymised and the dataset used didn ot contain supplementary information/videos for each trial. This is one of the limitations to our study. Another limitation to this study, also caused by the anonymisation of the dataset, was the inability to test whether there is a significant difference in performance between individualised models (models that are subject-specific and need to be trained on data from the user of the exoskeleton) and generalised models (models that are subject-independent and make predictions without the need to be trained on data from a specific user). This is an important point to consider when designing exoskeleton control strategies.

## 5. Conclusions

To conclude, we have used two deep learning models, LSTM and CNN, to forecast the trajectories of children with neurological disorders. The results show that our deep learning models outperform the three baseline methods we implemented in our study (with the LSTM being the top performer), with only two exceptions in which the FCN was better (see Section 3.5 for details). We also experimented with varying input and output windows to quantify how the performance is affected by the amount of input data and the length of the future horizon. A potential application of our approach is the control of lower limb robotics, whereby forecasted trajectories of the models can be used as a proxy for the user’s intentions. These intentions can be integrated into the control hierarchy of exoskeletons, specifically into the high-level control responsible for detecting the user’s intention and passing it on to the mid and low levels to generate appropriate movement commands. For real-time systems, there is always a trade-off between performance and speed. Input windows therefore need to be large enough to achieve acceptable errors, but not too large to slow the system down. As future work, we should evaluate the performance of the models on data collected using wearable sensors (e.g., IMUs and foot pressure sensors) rather than motion capture systems. We should also evaluate the difference between the performance of individualised and generalised models. Furthermore, we believe that the forecasted trajectories need to be paired with a corrective algorithm, unique to every gait sequence. In this scenario, the user intention (coming from a trajectory forecasting model) is adjusted by a corrective algorithm that produces the ’desired trajectory’ used by the mid- and low-level controllers of the exoskeleton.

## Figures and Tables

**Table 1:** Hyper-parameter optimisation for the LSTM network, CNN, and FCN.

	Hyper-Parameter	Search Space	Selected Value
LSTM	learning rate	[0.1, 0.01, 0.001, 0.0001, 0.00001]	0.001
	number of LSTM layers	[1, 2, 3, 4]	4
	number of LSTM hidden units	[16, 32, 64, 100, 128]	128
CNN	learning rate	[0.1, 0.01, 0.001, 0.0001, 0.00001]	0.0001
	conv1D layer 1 channels	[16, 32, 48]	32
	conv1D layer 2 channels	[32, 48, 64]	48
	conv1D layer 3 channels	[64, 128, 256]	256
	conv1D layer 4 channels	[128, 256, 512]	512
	kernel size for layers 1, 2	[1, 2, 3, 4, 5, 6, 7]	7
	kernel size for layers 3, 4	[1, 2, 3, 4, 5, 6, 7]	7
	padding	[0, 1, 2, 3, 4, 5]	4
	conv1D stride	[1, 2, 3, 4, 5]	1
	dilation	[1, 2, 4]	1
FCN	learning rate	[0.1, 0.01, 0.001, 0.0001, 0.00001]	0.001
	hidden layers	[1, 2, 3, 4, 6, 8, 10]	3
	nodes per layer	[10, 20, 40, 60, 100, 140, 160, 200]	200

**Table 2:** Performance of LSTM in forecasting gait trajectories for varying input and output window sizes.

Input Window Size (ms)	Input Time- Steps	Output Window Size (ms)	Output Time- Steps	MSE (Degrees)	MSE std (Degrees)	MAE (Degrees)	MAE std (Degrees)	Mean Pearson Correlation Coefficient
50	6	8.33	1	0.034	0.065	0.143	0.115	1.000
100	12	8.33	1	0.077	0.130	0.214	0.177	1.000
200	24	8.33	1	0.027	0.055	0.126	0.105	1.000
**400**	**48**	**8.33**	**1**	**0.019**	**0.161**	**0.095**	**0.099**	**1.000**
600	72	8.33	1	0.030	0.266	0.126	0.119	1.000
800	96	8.33	1	0.020	0.125	0.107	0.092	1.000
1000	120	8.33	1	0.022	0.318	0.109	0.098	1.000
50	6	25	3	0.079	0.526	0.175	0.220	1.000
100	12	25	3	0.077	0.474	0.187	0.206	1.000
200	24	25	3	0.079	0.793	0.176	0.218	1.000
**400**	**48**	**25**	**3**	**0.080**	**3.068**	**0.169**	**0.227**	**1.000**
600	72	25	3	0.092	2.597	0.173	0.250	1.000
800	96	25	3	0.104	1.279	0.200	0.252	1.000
1000	120	25	3	0.117	2.028	0.223	0.261	1.000
50	6	50	6	0.614	4.115	0.461	0.633	0.998
100	12	50	6	0.422	3.956	0.365	0.537	0.998
200	24	50	6	0.416	4.416	0.350	0.541	0.998
400	48	50	6	0.381	2.773	0.356	0.505	0.998
600	72	50	6	0.426	7.653	0.352	0.550	0.998
**800**	**96**	**50**	**6**	**0.363**	**3.377**	**0.332**	**0.502**	**0.998**
1000	120	50	6	0.405	5.414	0.359	0.526	0.998
50	6	100	12	3.548	15.749	1.104	1.526	0.984
100	12	100	12	3.310	15.545	1.058	1.480	0.985
200	24	100	12	3.200	14.790	1.008	1.478	0.985
400	48	100	12	3.279	30.567	1.007	1.505	0.985
600	72	100	12	2.723	14.993	0.927	1.365	0.987
800	96	100	12	2.524	13.366	0.906	1.305	0.988
**1000**	**120**	**100**	**12**	**2.157**	**9.940**	**0.847**	**1.200**	**0.990**
50	6	200	24	16.981	57.084	2.531	3.252	0.928
100	12	200	24	15.025	49.937	2.383	3.057	0.935
200	24	200	24	15.114	55.836	2.357	3.091	0.934
400	48	200	24	14.058	50.333	2.282	2.975	0.937
600	72	200	24	12.617	48.434	2.158	2.821	0.945
800	96	200	24	10.389	38.372	1.973	2.549	0.955
**1000**	**120**	**200**	**24**	**8.971**	**36.862**	**1.828**	**2.373**	**0.961**

Bold entries denote the lowest MSE and MAE values for a given output window size.

**Table 3:** Performance of CNN in forecasting gait trajectories for varying input and output window sizes.

Input Window Size (ms)	Input Time- Steps	Output Window Size (ms)	Output Time- Steps	MSE (Degrees)	MSE std (Degrees)	MAE (Degrees)	MAE std (Degrees)	Mean Pearson Correlation Coefficient
50	6	8.33	1	0.069	0.960	0.129	0.229	1.000
50	6	25	3	0.184	1.699	0.234	0.360	0.999
50	6	50	6	0.891	4.685	0.552	0.766	0.996
50	6	100	12	5.265	20.277	1.358	1.850	0.977
50	6	200	24	20.437	65.596	2.840	3.517	0.913
1000	120	8.33	1	0.061	0.709	0.138	0.203	1.000
1000	120	25	3	0.216	2.020	0.259	0.386	0.999
1000	120	50	6	0.994	4.966	0.576	0.814	0.995
1000	120	100	12	5.496	18.724	1.440	1.850	0.975
1000	120	200	24	18.007	54.453	2.738	3.242	0.926

**Table 4:** Benchmarking performance of deep learning models.

Input Window Size (ms)	Input Time- Steps	Output Window Size (ms)	Output Time- Steps	LSTM MAE (Degrees)	CNN MAE (Degrees)	FCN MAE (Degrees)	Naïve Method 1 ${}^{a}$ MAE (Degrees)	Naïve Method 2 ${}^{b}$ MAE (Degrees)
50	6	8.33	1	0.143 *	0.129 *${}^{,†}$	0.195 *${}^{,†}$	0.449 ${}^{†}$	1.513 *${}^{,†}$
50	6	25	3	0.175 *	0.234 *${}^{,†}$	0.294 *${}^{,†}$	0.888 ${}^{†}$	1.916 *${}^{,†}$
50	6	50	6	0.461 *	0.552 *${}^{,†}$	0.568 *${}^{,†}$	1.517 ${}^{†}$	2.486 *${}^{,†}$
50	6	100	12	1.104 *	1.358 *${}^{,†}$	1.336 *${}^{,†}$	2.640 ${}^{†}$	3.494 *${}^{,†}$
50	6	200	24	2.531 *	2.840 *${}^{,†}$	2.489 *${}^{,†}$	4.417 ${}^{†}$	5.096 *${}^{,†}$
1000	120	8.33	1	0.109 *	0.138 *${}^{,†}$	0.369 *${}^{,†}$	0.448 ${}^{†}$	6.090 *${}^{,†}$
1000	120	25	3	0.223 *	0.259 *${}^{,†}$	0.594 *${}^{,†}$	0.888 ${}^{†}$	6.121 *${}^{,†}$
1000	120	50	6	0.359 *	0.576 *${}^{,†}$	0.902 *${}^{,†}$	1.520 ${}^{†}$	6.167 *${}^{,†}$
1000	120	100	12	0.847 *	1.440 *${}^{,†}$	1.454 *${}^{,†}$	2.651 ${}^{†}$	6.254 *${}^{,†}$
1000	120	200	24	1.828 *	2.738 *${}^{,†}$	2.320 *${}^{,†}$	4.448 ${}^{†}$	6.385 *${}^{,†}$

^*a*^ Naïve Method 1: all output time-steps are predicted to be the value of the last input time-step. ^*b*^ Naïve Method 2: all output time-steps are predicted to be the mean value of the input time-steps. * and ^†^ represent statistical significance compared to Naïve Method 1 and LSTM, respectively. Significance is based on pairwise *t*-tests (*p* < 0.05). Bold entries denote the lowest MAE value for a given input and output window size.

## Data Availability

The dataset used in this study has been collected by Gillette Speciality Healthcare, and publicly shared at https://simtk.org/frs/?group_id=1946 (accessed on 1 October 2021).

## Abbreviations

The following abbreviations are used in this manuscript:

LSTM	Long Short-Term Memory
CNN	Convolutional Neural Network
FCN	Fully Connected Network
MAE	Mean Absolute Error
MSE	Mean Squared Error
